# Development of a machine learning-based clinical decision support system to predict clinical deterioration in patients visiting the emergency department

**DOI:** 10.1038/s41598-023-35617-3

**Published:** 2023-05-26

**Authors:** Arom Choi, So Yeon Choi, Kyungsoo Chung, Hyun Soo Chung, Taeyoung Song, Byunghun Choi, Ji Hoon Kim

**Affiliations:** 1 Department of Emergency Medicine, Yonsei University College of Medicine, 50 Yonsei-ro, Seodaemun-gu, Seoul, 03722 Republic of Korea; 2 Division of Pulmonary and Critical Care Medicine, Department of Internal Medicine, Yonsei University College of Medicine, 50 Yonsei-ro, Seodaemun-gu, Seoul, 03722 Republic of Korea; 3 Institute for Innovation in Digital Healthcare, Yonsei University, 50 Yonsei-ro, Seodaemun-gu, Seoul, 03722 Republic of Korea; 4 LG Electronics, 128 Yeoui-daero, Yeongdeungpo-gu, Seoul, 07336 Republic of Korea

**Keywords:** Computational models, Data integration

## Abstract

This study aimed to develop a machine learning-based clinical decision support system for emergency departments based on the decision-making framework of physicians. We extracted 27 fixed and 93 observation features using data on vital signs, mental status, laboratory results, and electrocardiograms during emergency department stay. Outcomes included intubation, admission to the intensive care unit, inotrope or vasopressor administration, and in-hospital cardiac arrest. eXtreme gradient boosting algorithm was used to learn and predict each outcome. Specificity, sensitivity, precision, F1 score, area under the receiver operating characteristic curve (AUROC), and area under the precision-recall curve were assessed. We analyzed 303,345 patients with 4,787,121 input data, resampled into 24,148,958 1 h-units. The models displayed a discriminative ability to predict outcomes (AUROC > 0.9), and the model with lagging 6 and leading 0 displayed the highest value. The AUROC curve of in-hospital cardiac arrest had the smallest change, with increased lagging for all outcomes. With inotropic use, intubation, and intensive care unit admission, the range of AUROC curve change with the leading 6 was the highest according to different amounts of previous information (lagging). In this study, a human-centered approach to emulate the clinical decision-making process of emergency physicians has been adopted to enhance the use of the system. Machine learning-based clinical decision support systems customized according to clinical situations can help improve the quality of care.

## Introduction

Clinical decision support systems (CDSS) contribute to patient safety and improve clinical outcomes^1,2^. Machine learning (ML) is being widely used in CDSS owing to its usefulness in diagnosis, prognosis, pattern recognition, and imaging classification with profound processing speed and the comprehensive nature of analytic methods^3^. The emergency care domain is particularly suitable for the challenge of adopting ML-based CDSS, because of the need for rapid clinical decision-making by physicians^4^. Accordingly, attempts at developing ML-based CDSS that enable efficient prediction for clinical practice have been reported in the setting of emergency departments (EDs)^3,5–7^.

Globally, increased ED visits have led to resource saturation and crowding^8^, which affects both physicians and patients^9^. Workflows are delayed in frequently crowded EDs^10–13^, making patients requiring time-critical interventions vulnerable to worse outcomes^14–16^. Therefore, it is crucial for CDSS to be able to assist the ED physicians who make time-critical decisions and interventions^4^. However, previous studies have been limited to a narrow range of input data and were inappropriate for ED use since they dealt with broad and prolonged outcomes^5,7^. Additionally, the validation of ML-based CDSS remains challenging in clinical practice^17^.

The trust of physicians in ML-based CDSS is essential for their application in clinical practice, which can be achieved through explainability for clinical relevance^17,18^. Moreover, CDSS allow ED physicians as end-users to provide tailored control, specific to their clinical use. Nevertheless, the interpretability of ML-based CDSS is insufficient in their ED application. Limited studies have reflected the sequential processing of ED management^3^.

Therefore, we aimed to develop a practical ML-based CDSS for ED practice utilizing accessible clinical data according to the decision-making framework of physicians and to validate its clinical usefulness as a supportive tool in the ED.

## Methods

### Study design and setting

We conducted a retrospective, observational study using data from a Level 1 ED of a tertiary teaching hospital in South Korea from June 2015 to December 2019. By law, level 1 and 2 EDs must have 24 h/day staffing by board-certified emergency physicians^19^. During the study, an average of approximately 8000 adult patients visited the ED per month at the study site. Approximately 21% of the patients were admitted to the hospital from the ED, and an average of 2.5% patients were admitted to intensive care units (ICU) per month.

Upon arrival in this ED, vital signs and illness severity were assessed by a qualified triage nurse, and patients were shifted to appropriate treatment areas. The adult ED is divided into four different treatment areas: (1) 13 beds for patients who are extremely unstable and/or require intensive care and close monitoring, (2) 26 beds for patients who are medically stable but require continuous monitoring, (3) 20 beds for stable patients without monitoring, (4) and a fast track for simple evaluation or treatment. This study complied with the tenets of the Declaration of Helsinki and was approved by the Institutional Review Board of Severance Hospital Human Research Protection Center, with a waiver for informed consent owing to its retrospective design and minimal harm to the patients (no. 4-2019-0555).

### Selection of participants

Patients aged > 18 years who visited the ED were included. Patients with missing basic information, those who died on arrival, and those without any laboratory testing in the ED where clinical monitoring was deemed unnecessary or who decided to get discharged following simple and rapid treatment were excluded.

### Data collection

Data were collected using a clinical research analysis portal system at the hospital’s Digital Healthcare Department. Randomized identification numbers for research were used for each patient to anonymously extract clinical data. Data on the following variables were collected for all patients who visited the ED: age, sex, visit methods, traumatic or non-traumatic causes of visit, medical history such as hypertension, diabetes mellitus, tuberculosis, hepatitis, allergies and operation history, chief complaints and their duration, Korean Triage and Acuity Scale score, systolic blood pressure (SBP), diastolic blood pressure (DBP), heart rate (HR), body temperature (BT), respiratory rate (RR), and mental status. Data were extracted from the electronic medical records by both physicians and nurses upon arrival, and 27 fixed features were composed as fixed values for each patient during the ED stay. Changes in SBP, DBP, HR, BT, RR, and mental status, consent for “do not attempt resuscitation” order obtained during the ED stay, laboratory test results, and electrocardiogram results at the ED were extracted from the test result records and nursing records and used to derive 93 observation features. For the outcome variables, we extracted the dispositional order for decision to ICUs by an ED physician, interventions such as inotropes and vasopressors, intubation for airway maintenance or mechanical ventilation, in-hospital cardiac arrest (IHCA) requiring cardiopulmonary resuscitation, and the time of their occurrence. Consequently, 120 features (27 fixed features and 93 observation features) and four outcome variables were processed to build the prediction model (Table 1).

**Table 1:** Summary of features.

Features	Category	Number	Description
Fixed	Patient information	6	Sex, age, visit method(2), reason of visit(2)
	Past medical histories	7	Hypertension, diabetes mellitus, pulmonary tuberculosis, hepatitis viral carrier, allergies, medications, operations
	Chief complaints and duration	6	Chief complaints categorized into 15 groups; cardiovascular, pulmonary/respiratory, gastrointestinal organs, gastrointestinal bleeding, neurologic, genitourinary, obstetric/gynecological, musculoskeletal, ophthalmologic, ear/nose/throat, skin, orofacial, psychiatric, medical device related, other general issues. If multiple complaints exist in each patient, up to 3 categories (3) and its duration(3) were used
	Vital signs on arrival	8	Systolic blood pressure(2), diastolic blood pressure(2), respiratory rate, pulse rate, oxygen saturation, body temperature
Observation	Clinical observation	6	Systolic blood pressure, diastolic blood pressure, respiratory rate, pulse rate, oxygen saturation, body temperature
	Mental status monitoring	1	Change of mental status; alert, drowsy, stuporous, semi-comatose, comatose, confused and sedated
	Electrocardiogram diagnosis	23	Coronary(5), electrophysiologic(12), metabolic (5), unspecified(1)
	Electrocardiogram metrics	8	P axes, R axes, T axes, PR interval, QRS duration, QT, QTc, ventricular rate
	Laboratory test	55	Arterial blood gas analysis result(12), regular laboratory results(43)

### Data pre-processing

Fixed features, observation features, and outcome variables were combined to create a single data table for the analysis of each participant. The study period ranged from ED arrival as the starting point to the occurrence of outcomes for the four variables, each as an endpoint, such that it created several data labels with different timelines following multiple outcomes for each patient. Additionally, to analyze the data over time, we resampled the data labels into units with intervals of 1 h. We pre-processed the resampled 1 h-units without any additional observation features using the carry-forward method to fill the unit with the closest values in chronological order. This approach is grounded in the assumption that until a physician detects a change in a patient's condition, the previous condition remains unchanged, and decisions are made based on previous data until the next random observation. Physicians make comprehensive decisions regarding the frequency of vital sign measurements, or the provision of immediate emergency intervention based on the patient's physical examination and previously measured vital signs.

**Figure 1:**
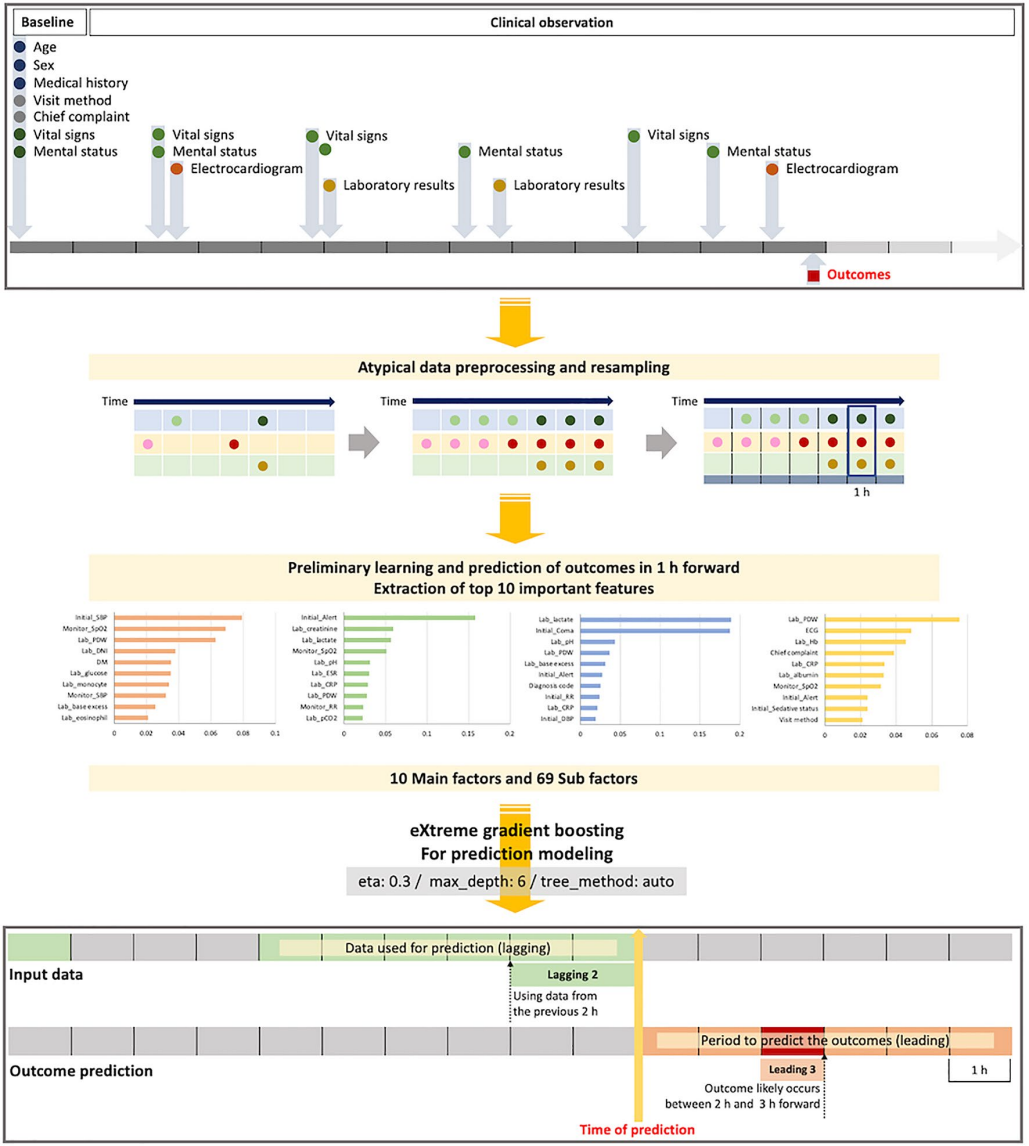
Data pre-processing and model development process.

For the outliers in vital sign data, we set the upper and lower limits of each variable considering the physiological range. Subsequently, the dataset was split into the training and test sets at 2:3 and 1:3 ratios, respectively, ensuring sufficient data for training and testing purposes. Owing to the imbalanced characteristics of the dataset, we performed randomized under-sampling in the training set to compensate for a considerably small proportion of outcomes. A dataset for each outcome was separately under-sampled using specific ratios. To verify the performance of the model in an environment similar to that of the real world, we did not apply under-sampling to the test set (Fig. 1).

### Model development

eXtreme gradient boosting (XGBoosting) was used to develop the model. XGBoosting is an algorithm with a combination of good prediction performance and fast processing time, compared with other ML algorithms. The term "gradient boosting" refers to the improvement of a single weak model by combining several weak models to collectively produce a powerful model that minimizes errors by setting goals for the following models. This algorithm is used in a wide range of applications, including regression, classification, ranking, and user-defined prediction, as it minimizes bias and underfitting^20^. First, we derived 10 important predictors that were highly associated with the outcome upon its occurrence. Next, we developed a baseline model to predict the occurrence of the outcome at the current time point (0 h) by weighting 10 important predictors and 69 subfactors. Based on this model, a total of 25 predictive models were developed that brought the input features up to the previous 1 h, 2 h, 3 h, and 6 h before (lagging), and predicted the occurrence of outcomes 1 h, 2 h, 3 h, and 6 h later (leading) from the time of prediction.

For example, in the case of inotropic use with leading 2 and lagging 3, the model predicted the use of inotropic medications between 1 and 2 h from the prediction time point using information collected in the previous 3 h (Fig. 1). The hyperparameters in each algorithm were tuned based on tenfold cross-validation during model development.

### Outcome measures

The outcome measures included IHCA, inotropic use, intubation, and ICU admission. The selection of the four indicators for clinical deterioration to be predicted in the ED clinical setting was informed by previous studies, which identified these outcomes as the most critical^21–24^. IHCA was defined as the development of witnessed or unwitnessed cardiac arrest with all causes within the ED. Inotropic use was defined as inotrope and vasopressor administration, such as noradrenaline, adrenaline, dopamine, dobutamine, or vasopressin, to overcome shock after adequate fluid administration. Intubation was defined as airway maintenance that protected the airway with positive pressure ventilation or conversion to a conventional ventilator from a home ventilator. ICU admission was defined as the physician’s order of admission to the ICU, regardless of the patient’s diagnosis.

### Analysis

Continuous data were expressed as mean values with standard deviations. Categorical data were expressed as frequencies and percentages. All tests were two-sided, with a statistical significance of P < 0.05. To assess the model’s performance, we used specificity, sensitivity, precision, F1 scores to assess the class imbalance, area under the receiver operating characteristic curve (AUROC), and area under the precision-recall curve (AUPRC). All statistical analyses were conducted with R Statistical Package (version 3.4.3) (www.R-project.org). Furthermore, we used XGBoosting (version 1.0.2) and Python (version 3.6.9, www.python.org)) programming environment for experiments and modeling^20,25^.

## Results

### Characteristics of study subjects

**Figure 2:**
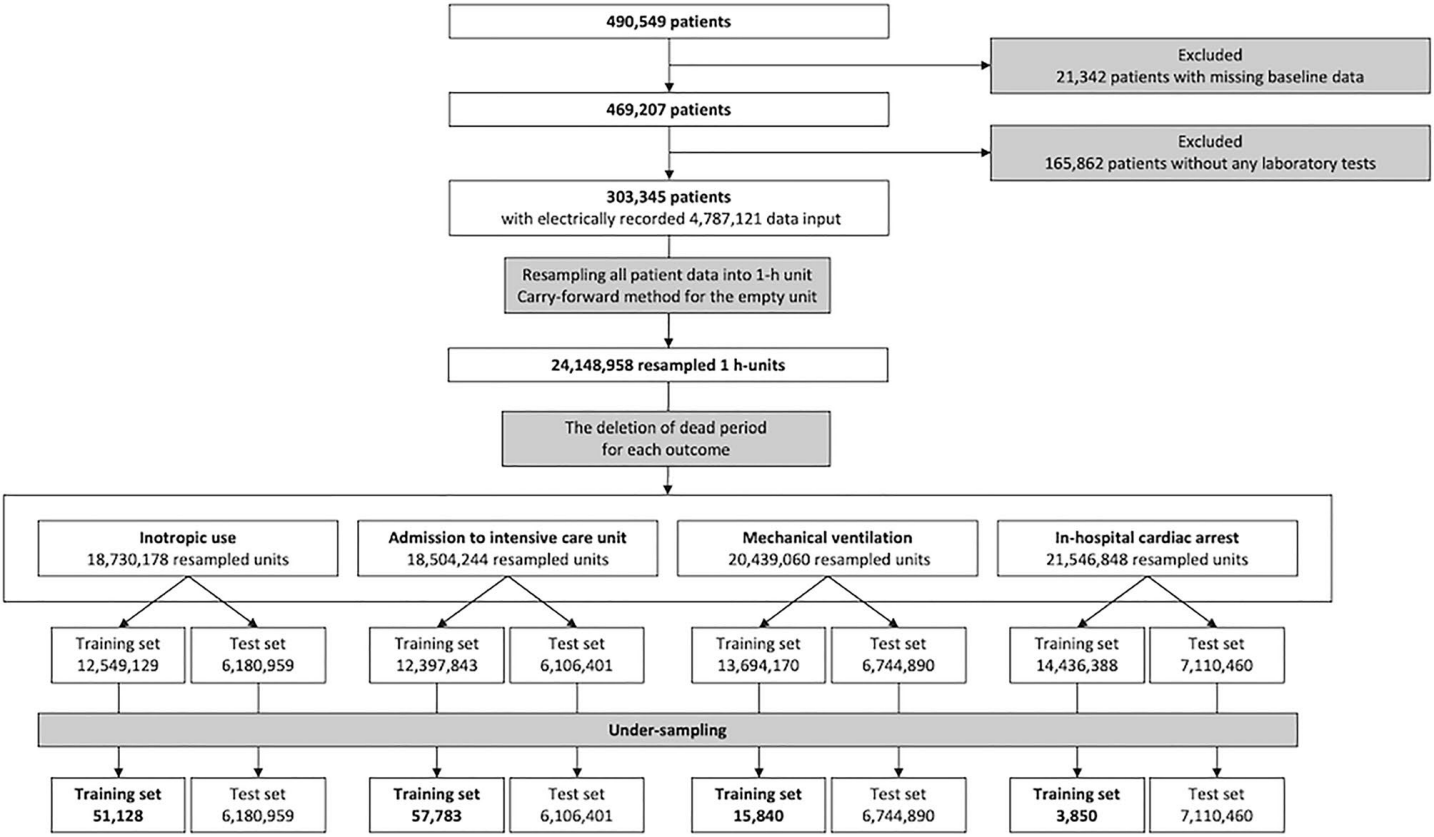
Flow chart for the study process.

Of the 490,549 patients, 21,342 were excluded because of missing basic information. A total of 469,207 patients were enrolled. Of these, 165,862 patients without laboratory tests were excluded. Eventually, we used the data of 303,345 patients with 4,787,121 input data, which were resampled into 24,148,958 resampled 1 h-units. This input dataset was split into a training set and a test set in 2:3 and 1:3 ratios, respectively, for each outcome (Fig. 2). Figure 2 depicts the data processing flow and the number of data handled. Table 2 summarizes the baseline characteristics of the study population.

**Table 2:** Baseline characteristics of the patients.

	Values	Missing data (%)
Age (mean ± SD)	52.21 ± 20.00	0
Sex (male), n (%)	142,208 (46.9)	0
Visit method (EMS), n (%)	76,497 (25.2)	0
Past medical history, n (%)		
Hypertension	28.54	1.35
Diabetes mellitus	15.68	1.35
Pulmonary tuberculosis	2.54	1.36
Hepatitis	3.2	1.37
Allergies	2.23	1.37
Operative history	23.96	1.48
Initial vital signs at arrival (mean ± SD)		
Systolic blood pressure (mmHg)	132.60 ± 26.60	1.45
Diastolic blood pressure (mmHg)	77.49 ± 14.72	1.45
Pulse rate (/min)	87.81 ± 18.66	1.44
Respiratory rate (/min)	17.03 ± 2.67	1.44
Oxygen saturation (%)	97.60 ± 7.42	2.62
Body temperature (°C)	36.78 ± 1.65	1.28
Mental status at arrival, n (%)		0
Alert	291,868 (96.2)	
Drowsy	5038 (1.7)	
Stupor	2361 (0.8)	
Semicoma	338 (0.1)	
Coma	468 (0.2)	
Sedated	3272 (1.1)	
Outcome, n (%)		
Intubation	2196 (0.7)	
Inotropic use	6939 (2.3)	
ICU admission	7914 (2.6)	
IHCA	543 (0.0)	

*SD* standard deviation, *EMS* emergency medical services, *ICU* intensive care unit, *IHCA* in-hospital cardiac arrest.

### Main results

**Figure 3:**
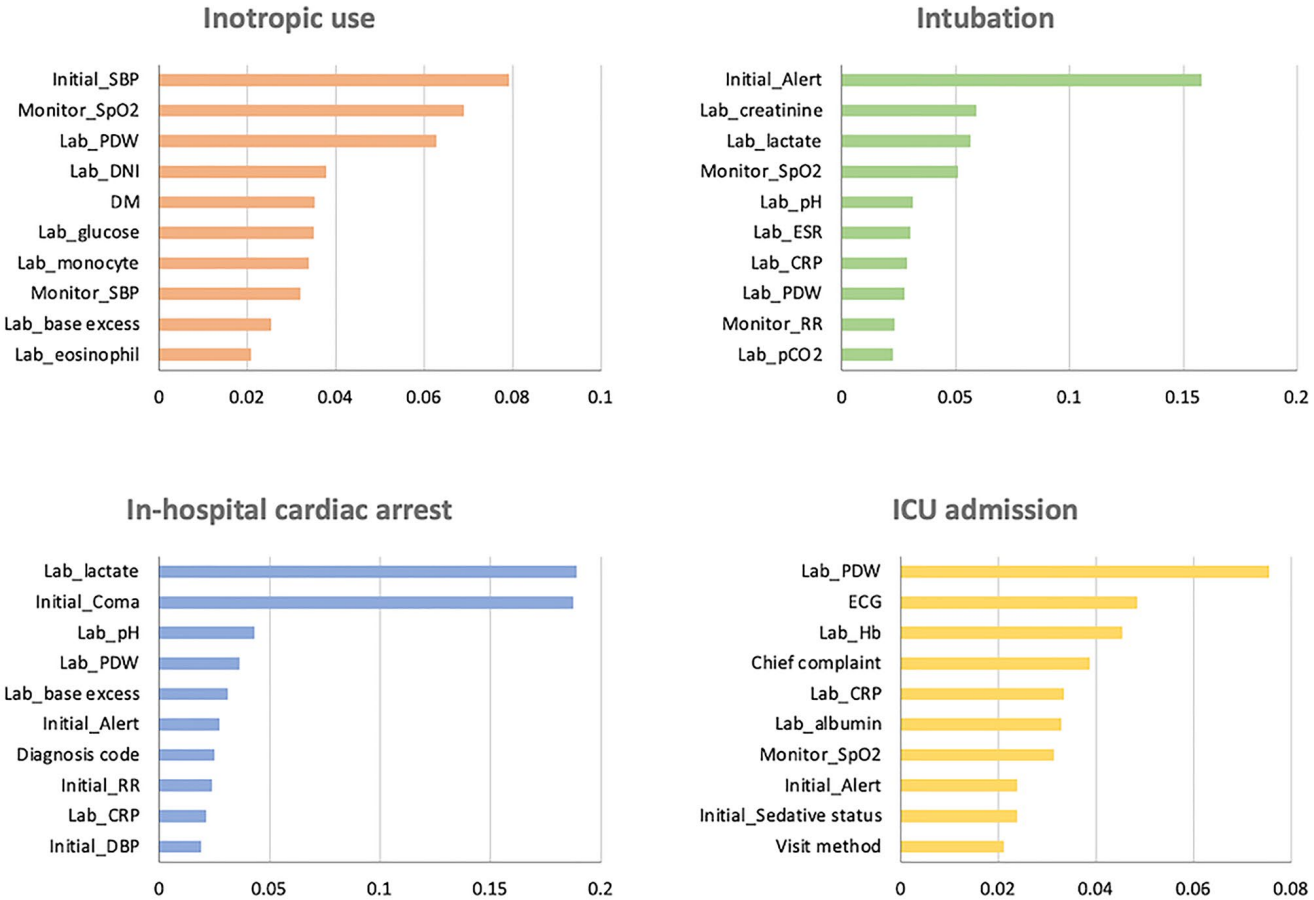
Top 10 important features in predicting each outcome. *ICU* intensive care unit, *SBP* systolic blood pressure, *SpO*_*2*_ oxygen saturation, *PDW* platelet distribution width, *DNI* delta neutrophil index, *DM* diabetes mellitus, *ESR* erythrocyte sedimentation rate, *CRP* C-reactive protein, *RR* respiratory rate, *DBP* diastolic blood pressure, *ICU* intensive care unit, *ECG* electrocardiogram, *Hb* hemoglobin.

Figure 3 depicts the 10 important predictors that were derived for the baseline predictive model for each outcome in the order of the feature importance score. The SBP upon arrival for inotropic use, alert mental status for intubation, lactate levels in IHCA, and platelet distribution width in ICU admission displayed the highest feature importance score.

**Figure 4:**
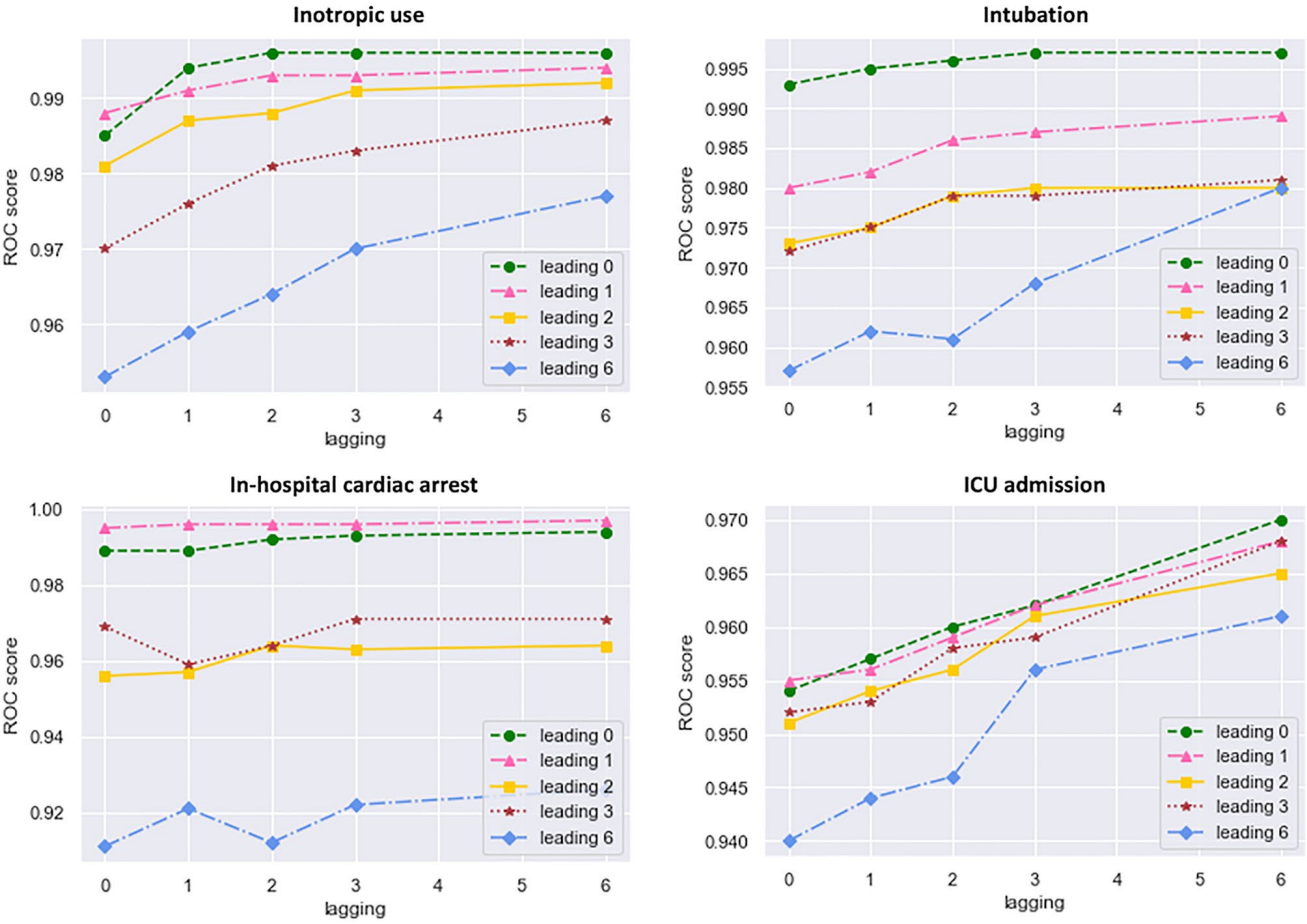
Area under receiver operating curve value for each outcome. *ROC* receiver operating curve, *ICU* intensive care unit.

The AUROC of the model with lagging 6 and leading 0 displayed the highest value among all models derived with lagging from 0 to 6 and leading 0 to 6 to predict all four outcomes (Fig. 4). Specifically, the AUROC increased upon acquiring more information from the past (lagging), except for predicting IHCA. The change in AUROC of IHCA was the smallest for all four outcomes with increased lagging. In cases with inotropic use, intubation, and ICU admission, the range of AUROC change with the leading 6 was the highest according to different amounts of previous information (lagging). Supplementary Table S1 summarizes the test characteristics, such as the specificity, recall, F1 score, precision, AUROC, and AUPRC, according to each leading and lagging for the four outcomes. Moreover, to enhance interpretability, we utilized explainable artificial intelligence with Shapley values for each model and provided the results in Supplementary Fig. S1, which illustrates how the model arrived at its predictions^26^. We subsequently performed external validation of the model using a separate dataset collected over a period of 2 years, and the results were shown in Supplementary Table S2 and Supplementary Fig. S2. Consistent with the findings of our internal validation, the external validation showed the same pattern, confirming our model’s comparable predictive power and generalizability.

**Figure 5:**
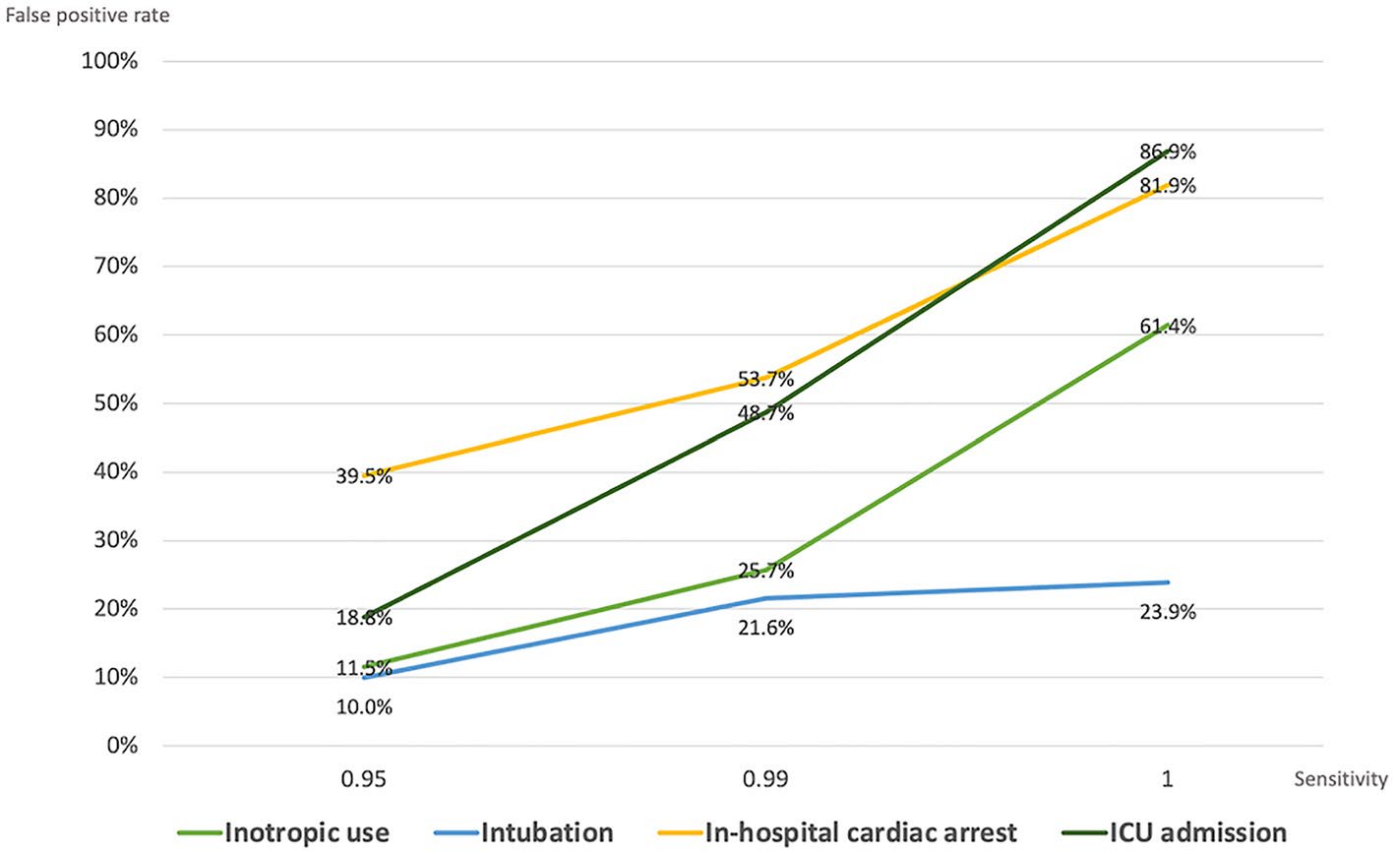
Fraction of false-positive cases in the model with leading 6 and lagging 6 for each outcome. *ICU* intensive care unit.

The number and percentage of false-positive cases with the sensitivity fixed at 95%, 99%, and 100% were analyzed to identify the accuracy with which the model predicted the deterioration of patients while allowing a certain level of false alarm. To predict inotropic use, 11.5% of false-positive cases with 95% sensitivity increased to 61.4% of cases with 100% sensitivity. Likewise, the false-positive rate increased from 10 to 23.9%, 39.5 to 81.9%, and 18.8 to 86.9% for the prediction of mechanical ventilation, in-hospital arrest, and ICU admission, respectively, upon changing the sensitivity of the predictive model from 95 to 100% (Fig. 5).

## Discussion

We developed a CDSS that helped ED physicians predict four critical deterioration events that should be detected pre-emptively and confirmed favorable prediction performances for each outcome. The prediction model developed in this study was designed to imitate the decision-making framework of an ED physician, which consists of three consecutive steps: observation, reasoning, and action^27^. Generally, physicians collect clinical findings from patients examined in the ED and perform reasoning based on their empirical expertise. Clinical findings during arrival occasionally change along with the random addition of novel findings during stays in the ED. Thus, physicians should perform additional reasoning to detect critical events after updating the clinical findings. Following this framework, we trained the model to recognize and reason with recent inputs by updating every hour with all clinical findings that were sequentially added or changed during the ED stay. Through this training, the overall AUROC of our models for the three critical events, except cardiac arrest, were increased when the leading was shorter (predicting the near future), and the lagging was longer (prediction based on more sequential information). In addition, in predicting events after 6 h, the longer the lagging was (the more sequential the information), the more the AUROC had improved. In other words, sequential information is more important for improving the accuracy of predicting events in the distant future. Previous studies that predicted critical events used several fixed variables, such as vital signs, as the predictors^17,28–31^. Contrarily, our prediction model was created by utilizing all clinical findings that occurred during ED practice and included laboratory tests and electrocardiograms, which have rarely been included as input variables in previous studies. Moreover, our study recognized updates in all clinical variables as recent observations. In practice, serially assessed clinical findings often contain missing values for various reasons and should be handled properly and efficiently^3^. Li et al. included patients with complete records to develop a machine-learning model to predict early mortality in the ED using electronic health records, potentially limiting the generalizability of their findings^32^. In contrast to this study, we assumed that ED physicians re-evaluated patients every hour and predicted critical events based on clinical findings in those reassessments. Therefore, the missing values observed in the 1 h-resampled unit were filled with the most recent values based on the physician's framework for decision-making. Thus, our model for ML-based CDSS was designed to imitate the sequential decision-making framework of ED physicians. Using all observations for referring to inferences in the real world, we attempted to induce ED physicians to use it in their practice. Moreover, we believe that our novel attempts can be extended to various clinical settings or outcome predictions with the same decision-making structure.

Models predicting clinical deterioration target two incompatible goals: the early detection of outcomes and fewer false-positive alerts to prevent alarm fatigue^33^. In the ED, it could be more fatal for a critical outcome to occur in patients without a CDSS-predicted deterioration than a false CDSS alarm. The failure to predict critical outcomes during ED practice is associated with unexpected death, and false alarms cause wastage of medical resources^34,35^. Unlike wards with relatively difficult access to human and material resources for critical patients, ED have easier access to these resources since they are staffed with resident medical personnel and equipped with resources for immediate resuscitation. Consequently, the negative impact of alarm fatigue is relatively less than that for other units. Therefore, we demonstrated a change in the number of false alarms after adjusting the sensitivity for predicting significant events from 95 to 100%. These data can be used as a reference by ED physicians in selecting an acceptable rate of false alarms that increases in proportion to accurate predictions of catastrophic events. Additionally, physicians supported by the ML-based CDSS can apply the optimal threshold for each lagging and leading based on practicality and the clinical environment. Therefore, the model was developed as a practical tool that could be customized per clinical unit.

Previous studies on the prediction of clinical deterioration in the ED have generally set outcomes with longer time windows, such as in-hospital mortality within 30 days^36^. In contrast, our study set prediction times of up to 6 h forward, that were appropriate to the ED. The reason for developing predictive models for the timely identification of patients at a high risk of clinical deterioration is to prioritize the point-of-care, effectively allocate resources, and prevent adverse outcomes^17^. However, previous ML-based predictive models have not been adequately tailored for context-specific patterns of care^37^. The primary priority of ED physicians is to pre-emptively detect critical events that require immediate interventions and to make resource-allocation decisions during a patient's stay in the ED^5,38^. Accordingly, we intended to develop a predictive model that could support physicians during point-of-care clinical decisions by presenting the outcomes within a relatively shorter time window. Since customization to the study setting is important for the feasibility of the predictive model, we set the time range based on the average ED length of stay in the study setting. Moreover, we determined three critical events requiring rapid resuscitation and ICU admission associated with a disposition decision were the most practical outcomes for developing an ML-based CDSS that supported ED physicians' practice.

For an application to the medical field, researchers should prepare ML-based CDSS for clinically actionable explanations. The physicians did not trust the predictions when the logic behind those was unclear^17,39–42^. We attempted to increase the explainability to physicians by presenting features that were highly influential in predicting the occurrence of the four critical events in our study and found that the features of the influence of each event were different. Additionally, we used an imbalanced dataset with a low event occurrence frequency. Therefore, we performed an under-sampling method and presented the results with AUROC and AUPRC as the performance metrics. AUPRC is a relative indicator because the baseline comprises the fraction of positives in the population^43^. The baseline AUPRC ranged from 0.000006 to 0.000424 according to each outcome, thereby representing an extremely small proportion of outcome occurrences in this study. However, the AUPRC in the present model showed superior values to the baseline AUPRC, with a maximum of 0.113, 0.234, 0.214, and 0.032 for cardiac arrest, inotropic use, intubation, and ICU admission, respectively.

The present study had several limitations. We selected the XGBoosting algorithm considering the study design using the tabular dataset because of its ease of use and overall good processing performance^44^. However, the process could be applied using other algorithms to develop predictive models that supposedly perform better. Second, the present study had a retrospective design, which presents a possibility of potential bias. Particularly, the medical staff manually updated the clinical findings of our dataset and did not record the findings that they were unaware of. This limitation can be compensated for by applying a real-time patient information acquisition system in clinical practice to obtain the dataset. Therefore, additional studies that prospectively evaluate the model using real-time datasets should be continued to prove the feasibility of our predictive model in daily ED practice. Third, the management of patients in a clinical setting may affect the study outcomes; however, our predictive model did not include this feature as an input. This necessitates expanding the modeling to include the clinical efforts of physicians in the future.

In summary, we developed a practical ML-based CDSS for ED practice by utilizing accessible clinical data according to the decision-making framework of physicians. CDSS should be customized according to clinical situations, and ML algorithms can help improve their performance.

## Supplementary Information

Supplementary Information.

## Data Availability

Data are available from the corresponding author upon reasonable request.
