# Supplementary material for: Development of a machine learning-based clinical decision support system to predict clinical deterioration in patients visiting the emergency department
Source: Sci Rep. 2023 May 26;13:8561. doi: 10.1038/s41598-023-35617-3 (PMC10220080; doi:10.1038/s41598-023-35617-3)
Supplement: Supplementary file 1 — Supplementary Information. [file 41598_2023_35617_MOESM1_ESM.docx]

**Supplementary Table S1. Test characteristics of the models in change of lagging with 0 h, 1 h, 2 h, 3 h, and 6 h in each outcome**

| **Outcome** | **Lagging** | **Leading** | **Specificity** | **Recall** | **F1 score** | **Precision** | **AUROC** | **AURPC** |
| --- | --- | --- | --- | --- | --- | --- | --- | --- |
| Inotropic use | **0** | 0 | 0.99 | 0.763 | 0.052 | 0.027 | 0.985 | 0.097 |
|  |  | 1 | 0.992 | 0.807 | 0.065 | 0.034 | 0.988 | 0.174 |
|  |  | 2 | 0.99 | 0.723 | 0.038 | 0.019 | 0.981 | 0.073 |
|  |  | 3 | 0.988 | 0.661 | 0.021 | 0.01 | 0.97 | 0.033 |
|  |  | 6 | 0.985 | 0.542 | 0.007 | 0.004 | 0.953 | 0.008 |
|  | **1** | 0 | 0.994 | 0.882 | 0.099 | 0.053 | 0.994 | 0.213 |
|  |  | 1 | 0.994 | 0.842 | 0.079 | 0.041 | 0.991 | 0.177 |
|  |  | 2 | 0.991 | 0.773 | 0.045 | 0.023 | 0.987 | 0.085 |
|  |  | 3 | 0.989 | 0.703 | 0.024 | 0.012 | 0.976 | 0.037 |
|  |  | 6 | 0.986 | 0.576 | 0.008 | 0.004 | 0.959 | 0.01 |
|  | **2** | 0 | 0.994 | 0.903 | 0.105 | 0.056 | 0.996 | 0.227 |
|  |  | 1 | 0.994 | 0.868 | 0.081 | 0.043 | 0.993 | 0.179 |
|  |  | 2 | 0.992 | 0.795 | 0.048 | 0.025 | 0.988 | 0.092 |
|  |  | 3 | 0.989 | 0.748 | 0.027 | 0.014 | 0.981 | 0.042 |
|  |  | 6 | 0.986 | 0.632 | 0.01 | 0.005 | 0.964 | 0.011 |
|  | **3** | 0 | 0.994 | 0.907 | 0.103 | 0.055 | 0.996 | 0.234 |
|  |  | 1 | 0.994 | 0.873 | 0.084 | 0.044 | 0.993 | 0.181 |
|  |  | 2 | 0.992 | 0.817 | 0.052 | 0.027 | 0.991 | 0.101 |
|  |  | 3 | 0.99 | 0.778 | 0.029 | 0.015 | 0.983 | 0.046 |
|  |  | 6 | 0.987 | 0.665 | 0.01 | 0.005 | 0.97 | 0.012 |
|  | **6** | 0 | 0.994 | 0.914 | 0.098 | 0.052 | 0.996 | 0.234 |
|  |  | 1 | 0.994 | 0.889 | 0.083 | 0.043 | 0.994 | 0.202 |
|  |  | 2 | 0.992 | 0.837 | 0.05 | 0.026 | 0.992 | 0.102 |
|  |  | 3 | 0.989 | 0.802 | 0.028 | 0.014 | 0.987 | 0.045 |
|  |  | 6 | 0.986 | 0.711 | 0.011 | 0.005 | 0.977 | 0.011 |
| Intubation | **0** | 0 | 0.995 | 0.852 | 0.035 | 0.018 | 0.993 | 0.161 |
|  |  | 1 | 0.993 | 0.749 | 0.013 | 0.007 | 0.98 | 0.047 |
|  |  | 2 | 0.99 | 0.663 | 0.006 | 0.003 | 0.973 | 0.017 |
|  |  | 3 | 0.989 | 0.603 | 0.004 | 0.002 | 0.972 | 0.004 |
|  |  | 6 | 0.987 | 0.599 | 0.002 | 0.001 | 0.957 | 0.001 |
|  | **1** | 0 | 0.996 | 0.876 | 0.041 | 0.021 | 0.995 | 0.195 |
|  |  | 1 | 0.993 | 0.77 | 0.013 | 0.007 | 0.982 | 0.061 |
|  |  | 2 | 0.99 | 0.702 | 0.006 | 0.003 | 0.975 | 0.013 |
|  |  | 3 | 0.989 | 0.617 | 0.004 | 0.002 | 0.975 | 0.003 |
|  |  | 6 | 0.988 | 0.605 | 0.002 | 0.001 | 0.962 | 0.002 |
|  | **2** | 0 | 0.996 | 0.886 | 0.042 | 0.022 | 0.996 | 0.214 |
|  |  | 1 | 0.993 | 0.791 | 0.015 | 0.008 | 0.986 | 0.061 |
|  |  | 2 | 0.99 | 0.738 | 0.006 | 0.003 | 0.979 | 0.015 |
|  |  | 3 | 0.989 | 0.645 | 0.004 | 0.002 | 0.979 | 0.004 |
|  |  | 6 | 0.988 | 0.592 | 0.002 | 0.001 | 0.961 | 0.002 |
|  | **3** | 0 | 0.996 | 0.897 | 0.042 | 0.022 | 0.997 | 0.212 |
|  |  | 1 | 0.993 | 0.812 | 0.014 | 0.007 | 0.987 | 0.071 |
|  |  | 2 | 0.99 | 0.755 | 0.006 | 0.003 | 0.98 | 0.019 |
|  |  | 3 | 0.989 | 0.668 | 0.004 | 0.002 | 0.979 | 0.004 |
|  |  | 6 | 0.987 | 0.633 | 0.002 | 0.001 | 0.968 | 0.002 |
|  | **6** | 0 | 0.995 | 0.897 | 0.04 | 0.02 | 0.997 | 0.207 |
|  |  | 1 | 0.992 | 0.833 | 0.013 | 0.007 | 0.989 | 0.055 |
|  |  | 2 | 0.99 | 0.752 | 0.006 | 0.003 | 0.98 | 0.016 |
|  |  | 3 | 0.989 | 0.636 | 0.004 | 0.002 | 0.981 | 0.004 |
|  |  | 6 | 0.988 | 0.605 | 0.002 | 0.001 | 0.98 | 0.002 |
| IHCA | **0** | 0 | 0.997 | 0.814 | 0.012 | 0.006 | 0.989 | 0.074 |
|  |  | 1 | 0.995 | 0.824 | 0.006 | 0.003 | 0.995 | 0.055 |
|  |  | 2 | 0.995 | 0.631 | 0.003 | 0.001 | 0.956 | 0.007 |
|  |  | 3 | 0.99 | 0.593 | 0.001 | 0.001 | 0.969 | 0.001 |
|  |  | 6 | 0.988 | 0.545 | 0.001 | 0 | 0.911 | 0.001 |
|  | **1** | 0 | 0.997 | 0.797 | 0.011 | 0.006 | 0.989 | 0.093 |
|  |  | 1 | 0.995 | 0.824 | 0.006 | 0.003 | 0.996 | 0.06 |
|  |  | 2 | 0.994 | 0.607 | 0.002 | 0.001 | 0.957 | 0.008 |
|  |  | 3 | 0.99 | 0.576 | 0.001 | 0.001 | 0.959 | 0.001 |
|  |  | 6 | 0.987 | 0.591 | 0.001 | 0 | 0.921 | 0.001 |
|  | **2** | 0 | 0.996 | 0.797 | 0.01 | 0.005 | 0.992 | 0.091 |
|  |  | 1 | 0.995 | 0.838 | 0.006 | 0.003 | 0.996 | 0.055 |
|  |  | 2 | 0.993 | 0.631 | 0.002 | 0.001 | 0.964 | 0.006 |
|  |  | 3 | 0.989 | 0.627 | 0.001 | 0 | 0.964 | 0.001 |
|  |  | 6 | 0.987 | 0.614 | 0.001 | 0 | 0.912 | 0.001 |
|  | **3** | 0 | 0.996 | 0.814 | 0.011 | 0.005 | 0.993 | 0.093 |
|  |  | 1 | 0.994 | 0.875 | 0.006 | 0.003 | 0.996 | 0.055 |
|  |  | 2 | 0.991 | 0.655 | 0.002 | 0.001 | 0.963 | 0.005 |
|  |  | 3 | 0.987 | 0.644 | 0.001 | 0 | 0.971 | 0.001 |
|  |  | 6 | 0.987 | 0.591 | 0.001 | 0 | 0.922 | 0.001 |
|  | **6** | 0 | 0.996 | 0.855 | 0.009 | 0.005 | 0.994 | 0.113 |
|  |  | 1 | 0.993 | 0.897 | 0.005 | 0.003 | 0.997 | 0.046 |
|  |  | 2 | 0.988 | 0.643 | 0.001 | 0.001 | 0.964 | 0.004 |
|  |  | 3 | 0.987 | 0.576 | 0.001 | 0 | 0.971 | 0.002 |
|  |  | 6 | 0.983 | 0.682 | 0.001 | 0 | 0.926 | 0.003 |
| ICU admission | **0** | 0 | 0.982 | 0.57 | 0.026 | 0.013 | 0.954 | 0.02 |
|  |  | 1 | 0.981 | 0.578 | 0.024 | 0.012 | 0.955 | 0.018 |
|  |  | 2 | 0.981 | 0.569 | 0.022 | 0.011 | 0.951 | 0.016 |
|  |  | 3 | 0.982 | 0.547 | 0.018 | 0.009 | 0.952 | 0.014 |
|  |  | 6 | 0.983 | 0.479 | 0.011 | 0.006 | 0.94 | 0.007 |
|  | **1** | 0 | 0.983 | 0.58 | 0.027 | 0.014 | 0.957 | 0.024 |
|  |  | 1 | 0.982 | 0.58 | 0.025 | 0.013 | 0.956 | 0.02 |
|  |  | 2 | 0.982 | 0.597 | 0.023 | 0.012 | 0.954 | 0.02 |
|  |  | 3 | 0.982 | 0.574 | 0.02 | 0.01 | 0.953 | 0.015 |
|  |  | 6 | 0.983 | 0.506 | 0.012 | 0.006 | 0.944 | 0.009 |
|  | **2** | 0 | 0.983 | 0.624 | 0.029 | 0.015 | 0.96 | 0.025 |
|  |  | 1 | 0.983 | 0.603 | 0.027 | 0.014 | 0.959 | 0.022 |
|  |  | 2 | 0.982 | 0.602 | 0.023 | 0.012 | 0.956 | 0.021 |
|  |  | 3 | 0.982 | 0.595 | 0.021 | 0.01 | 0.958 | 0.017 |
|  |  | 6 | 0.983 | 0.536 | 0.013 | 0.006 | 0.946 | 0.009 |
|  | **3** | 0 | 0.983 | 0.621 | 0.03 | 0.015 | 0.962 | 0.028 |
|  |  | 1 | 0.983 | 0.617 | 0.027 | 0.014 | 0.962 | 0.023 |
|  |  | 2 | 0.983 | 0.635 | 0.026 | 0.013 | 0.961 | 0.021 |
|  |  | 3 | 0.983 | 0.613 | 0.022 | 0.011 | 0.959 | 0.018 |
|  |  | 6 | 0.983 | 0.542 | 0.013 | 0.006 | 0.956 | 0.01 |
|  | **6** | 0 | 0.983 | 0.683 | 0.032 | 0.017 | 0.97 | 0.032 |
|  |  | 1 | 0.982 | 0.667 | 0.029 | 0.015 | 0.968 | 0.025 |
|  |  | 2 | 0.982 | 0.675 | 0.026 | 0.013 | 0.965 | 0.025 |
|  |  | 3 | 0.982 | 0.679 | 0.023 | 0.012 | 0.968 | 0.019 |
|  |  | 6 | 0.983 | 0.61 | 0.014 | 0.007 | 0.961 | 0.01 |

AUROC, area under receiver operating characteristic curve; AUPRC, area under precision recall curve; IHCA, in-hospital cardiac arrest; and ICU, intensive care unit.

**Supplementary Figure S1. Shapley values with SHapley Additive exPlanation artificial intelligence for the models**


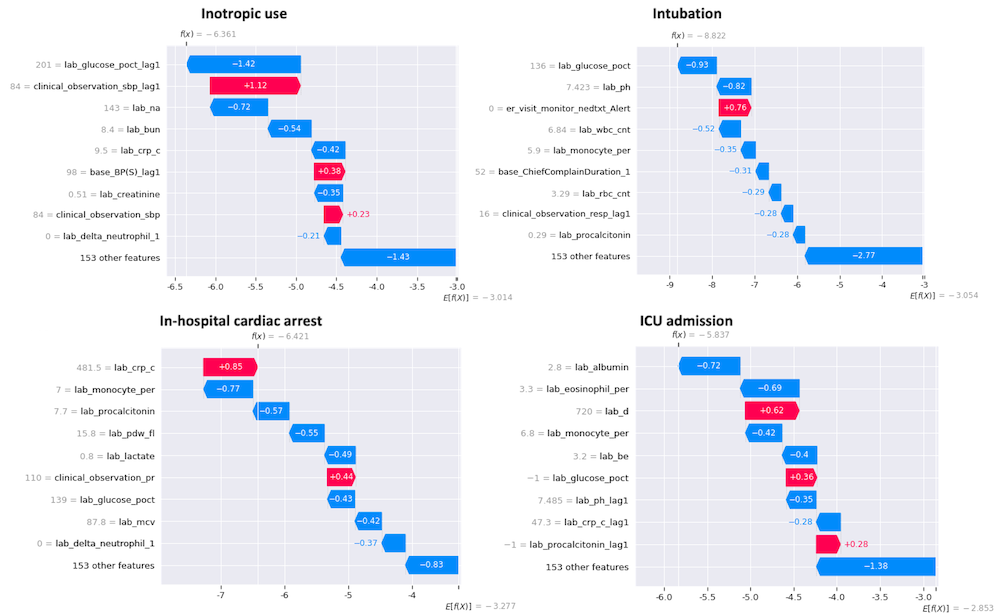


ICU, intensive care unit

**Supplementary Table S2. Test characteristics of the models with external validation**

| **Outcome** | **Lagging** | **Leading** | **Specificity** | **Recall** | **F1 score** | **Precision** | **AUROC** | **AURPC** |
| --- | --- | --- | --- | --- | --- | --- | --- | --- |
| Inotropic use | **0** | 0 | 0.989 | 0.549 | 0.036 | 0.018 | 0.943 | 0.058 |
|  |  | 1 | 0.99 | 0.607 | 0.035 | 0.018 | 0.958 | 0.06 |
|  |  | 2 | 0.991 | 0.521 | 0.028 | 0.014 | 0.947 | 0.032 |
|  |  | 6 | 0.988 | 0.394 | 0.007 | 0.004 | 0.907 | 0.005 |
|  | **1** | 0 | 0.994 | 0.733 | 0.081 | 0.043 | 0.978 | 0.108 |
|  |  | 1 | 0.992 | 0.685 | 0.05 | 0.026 | 0.973 | 0.081 |
|  |  | 2 | 0.992 | 0.607 | 0.036 | 0.019 | 0.961 | 0.047 |
|  |  | 6 | 0.988 | 0.448 | 0.009 | 0.004 | 0.923 | 0.006 |
|  | **3** | 0 | 0.995 | 0.794 | 0.097 | 0.051 | 0.985 | 0.126 |
|  |  | 1 | 0.993 | 0.773 | 0.068 | 0.036 | 0.983 | 0.102 |
|  |  | 2 | 0.993 | 0.709 | 0.048 | 0.025 | 0.976 | 0.058 |
|  |  | 6 | 0.989 | 0.581 | 0.012 | 0.006 | 0.956 | 0.01 |
|  | **6** | 0 | 0.993 | 0.839 | 0.086 | 0.045 | 0.988 | 0.125 |
|  |  | 1 | 0.993 | 0.809 | 0.064 | 0.033 | 0.986 | 0.1 |
|  |  | 2 | 0.992 | 0.768 | 0.045 | 0.023 | 0.981 | 0.06 |
|  |  | 6 | 0.989 | 0.638 | 0.013 | 0.007 | 0.963 | 0.012 |
| Intubation | **0** | 0 | 0.993 | 0.692 | 0.024 | 0.012 | 0.981 | 0.072 |
|  |  | 1 | 0.994 | 0.578 | 0.014 | 0.007 | 0.969 | 0.023 |
|  |  | 2 | 0.993 | 0.488 | 0.007 | 0.003 | 0.953 | 0.005 |
|  |  | 6 | 0.994 | 0.275 | 0.002 | 0.001 | 0.937 | 0.001 |
|  | **1** | 0 | 0.994 | 0.741 | 0.03 | 0.015 | 0.983 | 0.103 |
|  |  | 1 | 0.995 | 0.621 | 0.016 | 0.008 | 0.972 | 0.026 |
|  |  | 2 | 0.993 | 0.525 | 0.007 | 0.004 | 0.956 | 0.007 |
|  |  | 6 | 0.994 | 0.345 | 0.002 | 0.001 | 0.941 | 0.001 |
|  | **3** | 0 | 0.995 | 0.792 | 0.035 | 0.018 | 0.988 | 0.107 |
|  |  | 1 | 0.994 | 0.686 | 0.014 | 0.007 | 0.978 | 0.029 |
|  |  | 2 | 0.993 | 0.593 | 0.008 | 0.004 | 0.963 | 0.007 |
|  |  | 6 | 0.994 | 0.357 | 0.002 | 0.001 | 0.951 | 0.001 |
|  | **6** | 0 | 0.995 | 0.824 | 0.036 | 0.019 | 0.991 | 0.127 |
|  |  | 1 | 0.994 | 0.743 | 0.016 | 0.008 | 0.984 | 0.032 |
|  |  | 2 | 0.992 | 0.669 | 0.008 | 0.004 | 0.971 | 0.008 |
|  |  | 6 | 0.992 | 0.538 | 0.003 | 0.001 | 0.967 | 0.002 |
| IHCA | **0** | 0 | 0.995 | 0.757 | 0.01 | 0.005 | 0.984 | 0.095 |
|  |  | 1 | 0.992 | 0.447 | 0.002 | 0.001 | 0.946 | 0.002 |
|  |  | 2 | 0.995 | 0.41 | 0.002 | 0.001 | 0.946 | 0.002 |
|  |  | 6 | 0.991 | 0.302 | 0.0 | 0.0 | 0.911 | 0.002 |
|  | **1** | 0 | 0.994 | 0.783 | 0.01 | 0.005 | 0.986 | 0.13 |
|  |  | 1 | 0.994 | 0.497 | 0.003 | 0.001 | 0.949 | 0.003 |
|  |  | 2 | 0.993 | 0.515 | 0.002 | 0.001 | 0.952 | 0.002 |
|  |  | 6 | 0.992 | 0.321 | 0.0 | 0.0 | 0.911 | 0.0 |
|  | **3** | 0 | 0.995 | 0.817 | 0.012 | 0.006 | 0.991 | 0.113 |
|  |  | 1 | 0.995 | 0.553 | 0.003 | 0.002 | 0.962 | 0.003 |
|  |  | 2 | 0.992 | 0.575 | 0.002 | 0.001 | 0.97 | 0.003 |
|  |  | 6 | 0.99 | 0.358 | 0.0 | 0.0 | 0.928 | 0.0 |
|  | **6** | 0 | 0.995 | 0.841 | 0.011 | 0.006 | 0.994 | 0.077 |
|  |  | 1 | 0.994 | 0.604 | 0.003 | 0.002 | 0.969 | 0.003 |
|  |  | 2 | 0.99 | 0.634 | 0.002 | 0.001 | 0.965 | 0.002 |
|  |  | 6 | 0.991 | 0.415 | 0.001 | 0.0 | 0.958 | 0.001 |
| ICU admission | **0** | 0 | 0.993 | 0.317 | 0.016 | 0.008 | 0.915 | 0.007 |
|  |  | 1 | 0.987 | 0.367 | 0.021 | 0.011 | 0.913 | 0.01 |
|  |  | 2 | 0.985 | 0.375 | 0.022 | 0.011 | 0.911 | 0.012 |
|  |  | 6 | 0.98 | 0.36 | 0.017 | 0.009 | 0.887 | 0.009 |
|  | **1** | 0 | 0.993 | 0.334 | 0.018 | 0.009 | 0.92 | 0.009 |
|  |  | 1 | 0.985 | 0.403 | 0.02 | 0.01 | 0.921 | 0.011 |
|  |  | 2 | 0.985 | 0.4 | 0.023 | 0.012 | 0.911 | 0.013 |
|  |  | 6 | 0.989 | 0.288 | 0.024 | 0.012 | 0.895 | 0.011 |
|  | **3** | 0 | 0.993 | 0.497 | 0.024 | 0.012 | 0.943 | 0.015 |
|  |  | 1 | 0.985 | 0.489 | 0.024 | 0.012 | 0.93 | 0.016 |
|  |  | 2 | 0.984 | 0.494 | 0.027 | 0.014 | 0.938 | 0.018 |
|  |  | 6 | 0.985 | 0.42 | 0.026 | 0.013 | 0.91 | 0.017 |
|  | **6** | 0 | 0.99 | 0.679 | 0.024 | 0.012 | 0.961 | 0.019 |
|  |  | 1 | 0.983 | 0.655 | 0.028 | 0.015 | 0.956 | 0.022 |
|  |  | 2 | 0.977 | 0.687 | 0.027 | 0.014 | 0.958 | 0.026 |
|  |  | 6 | 0.974 | 0.599 | 0.022 | 0.011 | 0.93 | 0.018 |

AUROC, area under receiver operating characteristic curve; AUPRC, area under precision recall curve; IHCA, in-hospital cardiac arrest; and ICU, intensive care unit.

**Supplementary Figure S2. Area under receiver operating characteristic value for each outcome**

**
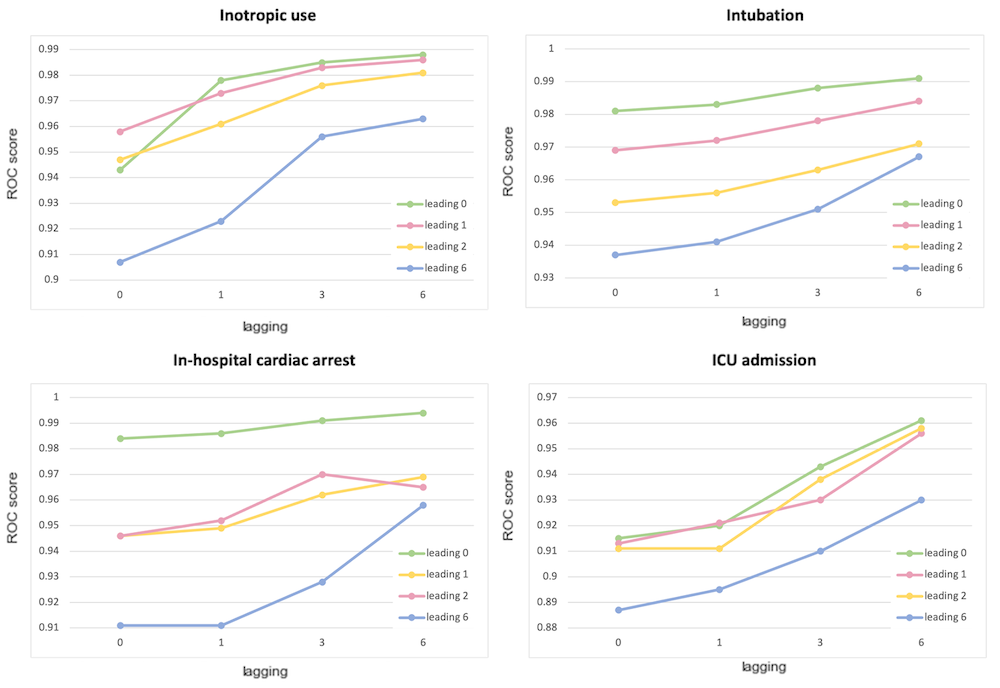
**

ROC, receiver operating characteristic curve; ICU, intensive care unit
